# A risk score for the prognosis prediction of the muscle-invasive bladder cancer patients who received gemcitabine plus cisplatin chemotherapy

**DOI:** 10.18632/aging.204424

**Published:** 2022-12-05

**Authors:** Yupeng Guo, Jing Dong, Tao Ji, Xiaoxia Li, Shengzhong Rong, Hongjun Guan

**Affiliations:** 1 Department of Epidemiology and Statistics, School of Public Health of Mudanjiang Medical University, Mudanjiang, Heilongjiang Province 157011, China

**Keywords:** bladder cancer, chemotherapy, gemcitabine, cisplatin, prognosis

## Abstract

To develop an individualized gene-based risk score to predict the prognosis of muscle-invasive bladder cancer (MIBC) patients who received GC regimens. We downloaded transcriptome profiling data and clinical information from the Cancer Genome Atlas (TCGA) database. We identified 1854 survival-associated genes and then constructed the risk score based on six special genes selected from the survival-associated genes. We divided patients into high-risk and low-risk groups according to the median risk score. High-risk patients have significantly poorer overall survival than low-risk patients (log-rank test chi-square = 38.08, *p* = 7e-10, C-index = 0.785, se = 0.032). The risk score was evaluated by Kaplan–Meier survival curve, time-dependent ROC curves, and C-index. Multivariate Cox regression and nomogram suggested that the risk score was an independent prognostic indicator. Gene set enrichment analysis indicated that the survival-associated genes were significantly enriched in immune-related terms. Among six special genes, CHPF2, TRAV26-2, and BTF3P12 were found to be immune-related genes. In conclusion, our risk score provided an indicator to predict the prognosis of MIBC patients who received GC regimens and potential immunotherapeutic targets for MIBC.

## INTRODUCTION

Bladder cancer (BC) is the ninth most common cancer worldwide. According to GLOBOCAN data, approximately 550,000 new cases were diagnosed in 2018, accounting for roughly 3% of all new cancer diagnoses worldwide [1]. In the United States (US), BC is the sixth most common cancer and the eighth most common cause of cancer death, with an estimated 80,500 newly diagnosed cases and an estimated 17,600 death in 2019 [2]. Although the incidence of BC has fallen over the past decades, the mortality of BC has remained steadfast since 1987 in the US [3].

Approximately 25% of all newly diagnosed BC patients have muscle-invasive bladder cancer (MIBC) every year [4]. MIBC treatment generally involves a combination of chemotherapy, radiation, and surgery. Radical cystectomy (RC) and pelvic lymph node dissection are the standard surgery approach. Still, many clinical trials showed patients would benefit more from additional chemotherapy by increasing five years overall survival (OS) and ten years OS by roughly 10%, respectively, than RC alone [5–10], and therefore chemotherapy plays an essential role in MIBC prognosis improvement. Within two decades, cisplatin-based neoadjuvant chemotherapy has become the standard approach for MIBC treatment. Compared with loco-regional therapy alone, cisplatin-based chemotherapy had a significant 5-year OS benefit and reduced risk of death [11, 12]. Gemcitabine and cisplatin (GC) regimens and methotrexate, vinblastine, adriamycin, and cisplatin (MVAC) regimens are the most common cisplatin-based regimens. Researchers and clinicians generally believed neither of the combinations was superior to the other in terms of progression and prognosis [13, 14], but new evidence suggested that patients who received MVAC regimens may have better survival outcomes than those who received GC regimens [12]. Many clinicians prefer the GC regimens due to their low toxicity and ease of administration [15] in clinical practice. However, many studies reported that 50% of MIBC patients are ineligible for cisplatin-based chemotherapy due to either age-related or disease-related risk comorbidities [4]. There is not a validated approach to predict the prognosis of MIBC patients who received cisplatin-based regimens yet, not to mention a similar study for the GC regimens.

This study aimed to develop an individualized gene-based risk score to predict the prognosis of MIBC patients who received GC regimens, verify its role in prognosis prediction and cisplatin-based regimens choice, and investigate its potential mechanisms.

## MATERIALS AND METHODS

### Clinical samples and data acquisition

We downloaded a transcriptome profiling dataset of 409 patients, a clinical characteristics dataset of 405 patients, and a drug treatment dataset of 427 patients from the BLCA project of the Cancer Genome Atlas (TCGA) database. As these three datasets contained different data, we merged three datasets into a study dataset that contained complete information about MIBC patients who received GC regimens. Before merging, we log2-transformed gene expression data in the transcriptome profiling dataset and converted nonstandard drug names to standardized drug names in the drug treatment dataset by querying the NCI Drug Dictionary (https://www.cancer.gov/publications/dictionaries/cancer-drug). As we had removed duplicate and unmatched patients during merging, the study dataset contained clinical characteristics information and gene expression data of 65 unduplicated MIBC patients.

### Analysis of survival-associated genes of the MIBC patients who received GC regimens

After converting gene expression data from the quantitative expression level to the relative expression level—high expression and low expression— by the median gene expression, we performed univariate cox regression analysis on the study dataset. We selected genes significantly associated with OS as the survival-associated gene for further analysis (adjusted *P*-value < 0.05). Overall survival time was defined by days from the first diagnosis to death or the last follow-up. Status ‘death’ meant the event happened and status’ last follow-up’ meant censored. We used Gene Ontology (GO) enrichment analysis to investigate the molecular functions of the survival-associated genes and Kyoto Encyclopedia of Genes and Genomes (KEGG) enrichment analysis to explore the potential molecular mechanisms of the survival-associated genes (adjusted *P*-value < 0.05). The survival-associated genes were annotated by the R package biomaRt [16]. GO enrichment analysis and KEGG enrichment analysis were performed using the R package clusterProfiler [17].

### Development of a risk score for the prognosis prediction of MIBC patients who received GC regimens

We selected a few special genes from the survival-associated genes by Least Absolute Shrinkage and Selection Operator (LASSO) cox regression analysis. Then we applied a multivariate cox regression analysis on these special survival-associated genes with Akaike’s information criterion (AIC). We calculated the individualized risk score of MIBC patients who received GC regimens by the formula generated from the multivariate cox regression analysis: $\text{risk score}=\sum_{i=1}^{n}\beta_{i}x_{i}$ where *x_i_* denoted the relative gene expression level and *β_i_* denoted the coefficient of the multivariate cox regression analysis. LASSO cox regression was performed using the R package glmnet [18].

### Evaluation of the predictive value of the risk score

We evaluated the predictive value of the risk score on the study dataset, where we considered its performance in the subsets of the clinical characteristics (age at diagnosis, gender, and ACJJ pathological stage). We divided the patients into high-risk and low-risk groups by the median risk score. Kaplan-Meier survival curve and log-rank test were employed to compare the OS of the high-risk and low-risk groups. Time-dependent receiver operating characteristic curve (ROC) was plotted to evaluate the predictive power of the risk score. The overall concordance statistic (C-index) was introduced to assess the predictive accuracy of the risk score. The relationship between the risk score and the clinical characteristics was investigated by comparing the average risk score level of different clinical characteristics subcategories and building a nomogram combing the risk score and the clinical characteristics.

### Analysis of differentially expressed genes (DEGs) between high-risk and low-risk groups in MIBC patients who received GC regimens

Differentially expressed genes (DEGs) between high-risk and low-risk groups were identified (|fold change| ≥ 2, *P*-value < 0.05). Gene Set Enrichment Analysis (GSEA) was employed to explore key signal pathways for the DEGs (adjust *P*-value < 0.05) based on C5: ontology gene sets (c5.all.v7.5.symbols.gmt, http://www.gsea-msigdb.org/gsea/msigdb/index.jsp) as the reference gene set. The DEGs were identified by R package edgeR [19], and GSEA was performed by R package clusterProfiler [17].

## RESULTS

**Figure 1:**
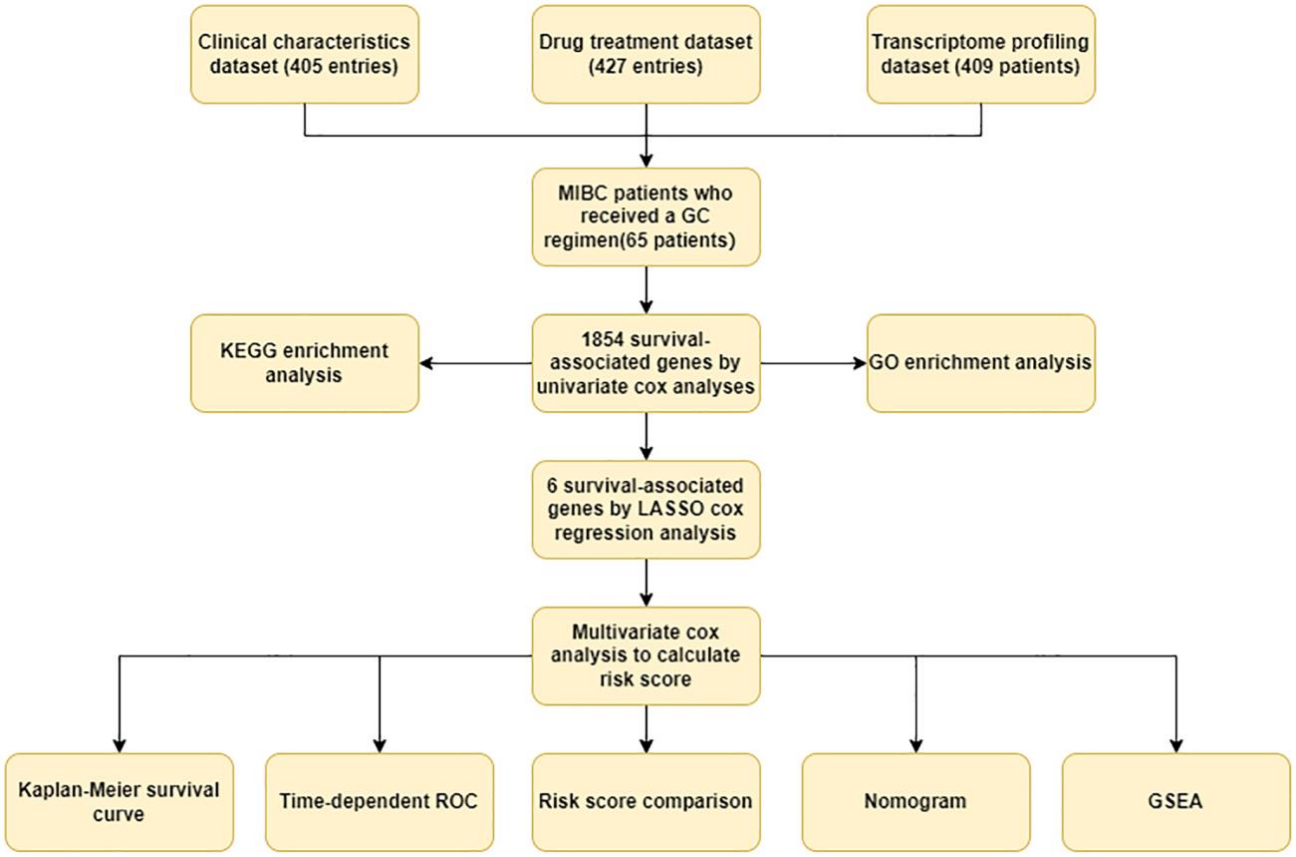
**Workflow of this study.** The clinical characteristics dataset, Drug treatment dataset, and transcriptome profiling dataset were downloaded from the BLCA (Bladder Urothelial Carcinoma) project of TCGA (The Cancer Genome Atlas). The study dataset contained complete information about 65 MIBC patients who received GC regimens, which was obtained by merging the clinical characteristics dataset, the Drug treatment dataset, and the transcriptome profiling dataset. Survival-associated genes were identified by univariate cox regression analysis. The KEGG enrichment analysis and the GO enrichment analysis were utilized to explore the molecular functions of the survival-associated genes. LASSO cox regression analysis was used to select special survival-associated genes. The individual risk score was calculated by multivariate cox regression analysis. Kaplan-Meier survival curve and log-rank test were employed to compare the OS of the high-risk and low-risk patients. To assess the risk score’s ability to predict prognosis, a time-dependent ROC plot was created. High-risk and low-risk MIBC patients’ average risk scores were compared. Risk score, gender, age at diagnosis, and AJCC pathological stage were all combined to create the nomogram. GSEA was utilized to explore key signal pathways for DEGs between high-risk and low-risk patients.

The workflow of this study is summarized in Figure 1.

### Clinical characteristics of MIBC patients who received GC regimens

A total of 65 MIBC patients were involved in this study. The clinical characteristics of the 65 MIBC patients are summarized in Table 1.

**Table 1:** Clinical characteristics of MIBC patients who received GC regimens.

**Clinical characteristics**	**Count**	**Percent (%)**
Gender		
Female	21	32.31
Male	44	67.69
Age at diagnosis (year)		
<65	34	52.31
≥65	31	47.69
AJCC pathological stage		
Stage II	17	26.15
Stage III	18	27.69
Stage IV	30	46.15
AJCC pathology T		
T2	3	5.77
T2b	5	9.62
T3	4	7.69
T3a	10	19.23
T3b	15	28.85
T4	2	3.85
T4a	12	23.08
T4b	1	1.92
AJCC pathology N		
N0	24	36.92
N1	12	18.46
N2	17	26.15
N3	1	1.54
NX	10	15.38
M0	23	35.38
AJCC pathology M		
M1	2	3.08
MX	40	61.54

### Identification of survival-associated genes of the MIBC patients who received GC regimens

A total of 1854 genes significantly associated with OS were identified as survival-associated genes, among which 1248 genes with a hazard ratio (HR) >1, indicating patients with a high expression level of these genes might have a poor survival time, and 606 genes with an HR <1, indicating patients with the high expression level of these genes might have a better survival time.

### Molecular functional analysis of the survival-associated genes

**Figure 2:**
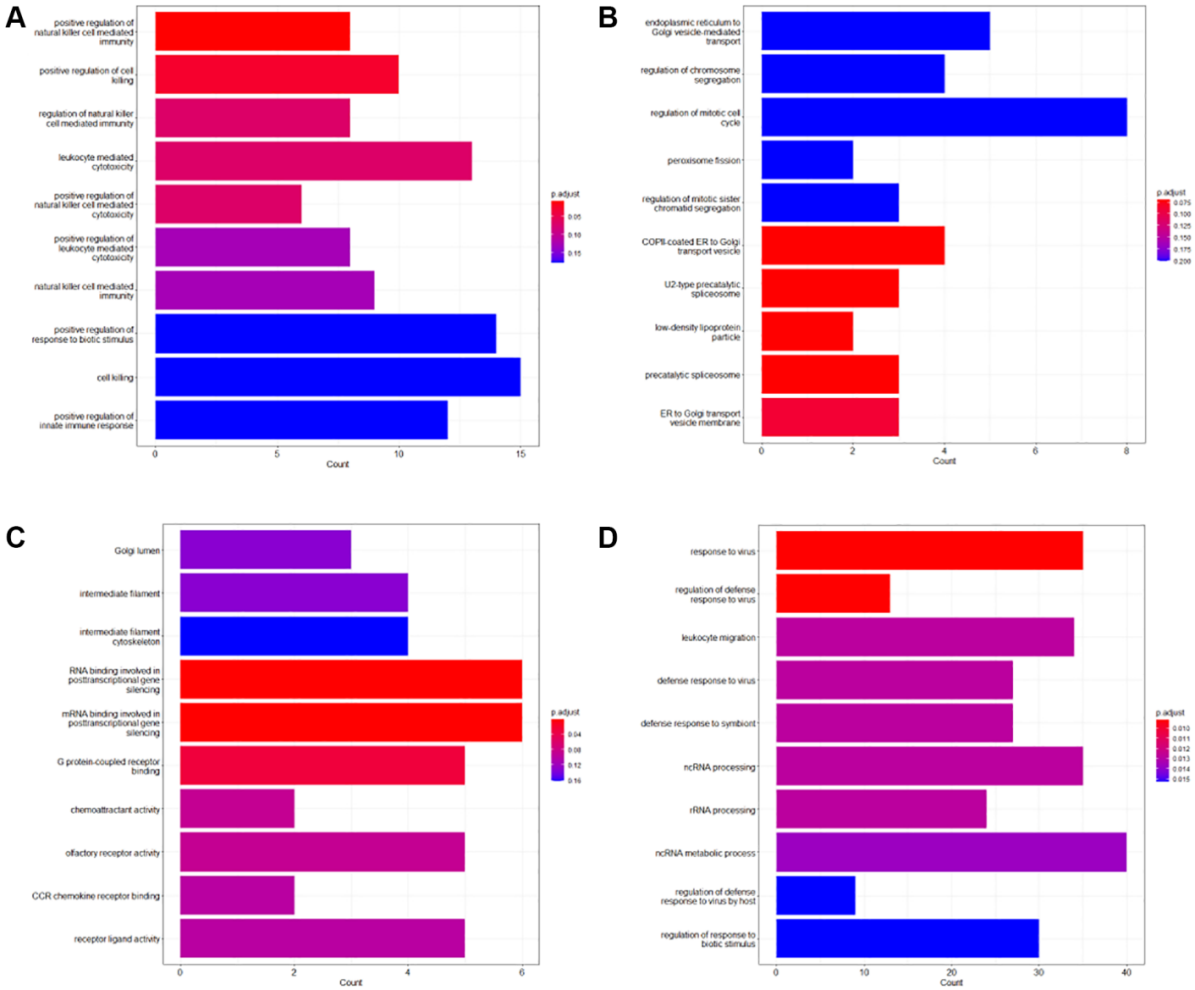
**GO enrichment analysis of the survival-associated genes.** (**A**) Top 10 GO enrichment terms of the survival-associated genes of the entire dataset; (**B**) Top 10 GO enrichment terms of the survival-associated genes of Stage II; (**C**) Top 10 GO enrichment terms of the survival-associated genes of Stage III; (**D**) Top 10 GO enrichment terms of the survival-associated genes of Stage IV.

**Figure 3:**
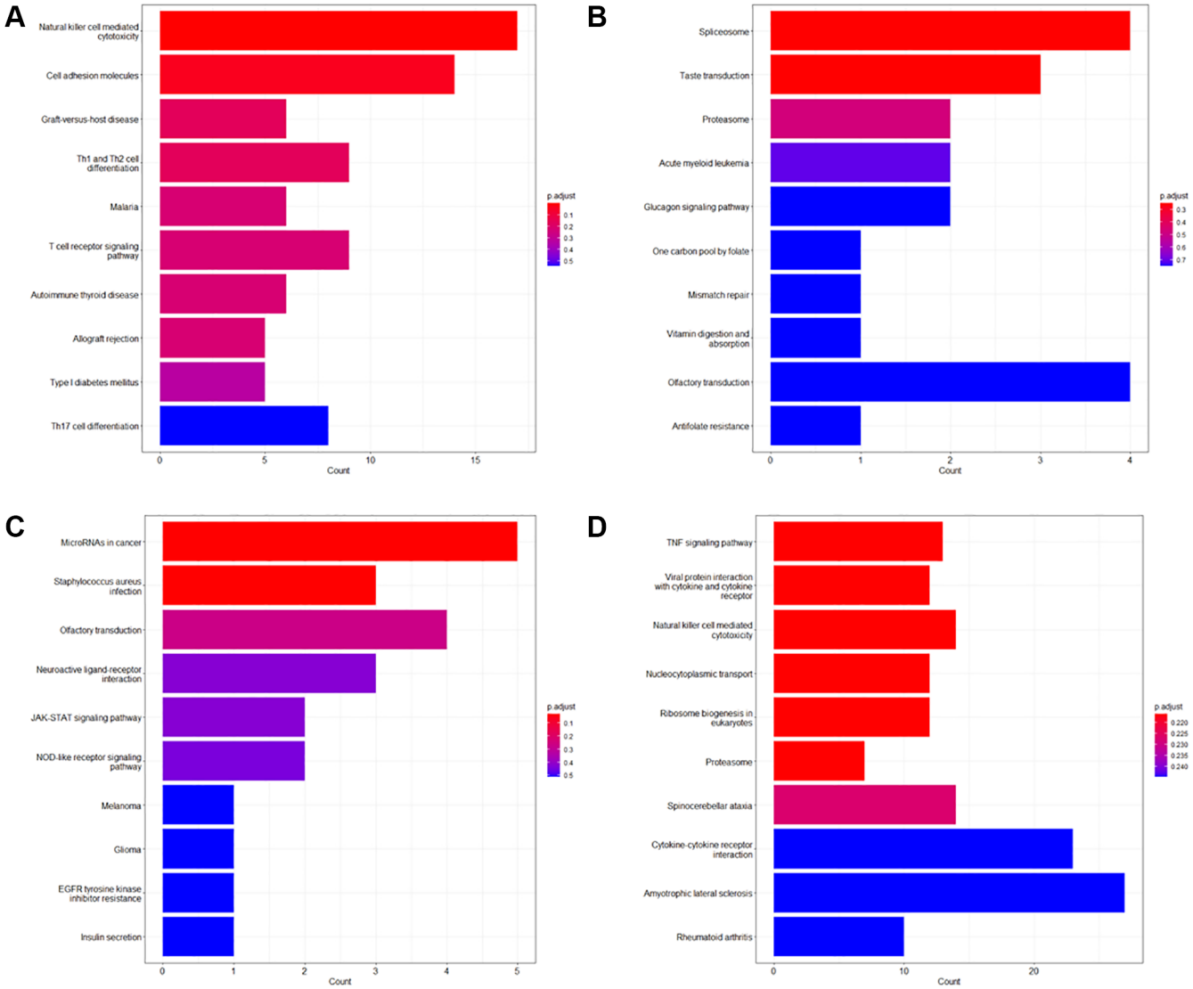
**KEGG enrichment analysis of the survival-associated genes.** (**A**) Top 10 KEGG enrichment terms of the survival-associated genes of the entire dataset; (**B**) Top 10 KEGG enrichment terms of the survival-associated genes of Stage II; (**C**) Top 10 KEGG enrichment terms of the survival-associated genes of Stage III; (**D**) Top 10 KEGG enrichment terms of the survival-associated genes of Stage IV.

The GO enrichment analysis showed that survival-associated genes were significantly enriched in ‘positive regulation of natural killer cell mediated immunity’ and ‘positive regulation of cell killing’ (adjust *P*-value < 0.05) terms. The KEGG enrichment analysis showed that survival-associated genes were significantly enriched in ‘Natural killer cell-mediated cytotoxicity’ and ‘Cell adhesion molecules’ pathways (adjust *P*-value < 0.05). We further performed GO enrichment analysis and KEGG enrichment analysis on the survival-associated genes of the patients in different ACJJ stages, and then several enrichment analyses ended with significant results. For ACJJ stage III, the survival-associated genes were significantly enriched in “RNA binding involved in posttranscriptional gene silencing”, “mRNA binding involved in posttranscriptional gene silencing”, and “G protein-coupled receptor binding” in GO enrichment analysis (adjust *P*-value < 0.05). In KEGG enrichment analysis, the survival-associated genes were significantly involved in regulating signaling pathways related to “MicroRNA in cancer” and “Staphylococcus aureus infection” (adjust *P*-value < 0.05). For stage IV, the survival-associated genes were significantly enriched in terms such as “response to virus”, “regulation of defense response to virus”, and “leukocyte migration” in the GO enrichment analysis (adjust *P*-value < 0.05) (Figures 2, 3).

### A risk score for the prognosis prediction of MIBC patients who received GC regimens

Twelve survival-associated genes were picked by LASSO cox regression analysis, where a leave-one-out cross-validation approach was utilized to tune the optimal parameters. These twelve survival-associated genes were subjected to multivariate cox regression analysis, and six genes were left in the model by AIC (Table 2). Based on the relative expression level of the selected six genes, we constructed a risk score which was calculated by the formula: risk score = (1.3056 × ENSG00000033100) + (−1.2720 × ENSG00000211812) + (1.8782 × ENSG00000213003) + (−1.9134 × ENSG00000231150) + (−1.0926 × ENSG00000236047) + (−2.1814 × ENSG00000239224), where the coefficients denoted the coefficient of the multivariate cox regression analysis. We used this formula to calculate the individualized risk score of each MIBC patient.

**Table 2:** Multivariate Cox regression analysis of the survival-associated genes.

**ENSEMBL**	**Symbol**	**coef**	**exp (coef)**	**se (coef)**	**z**	***P*-value**
ENSG00000033100	CHPF2	1.3056	3.6901	0.558	2.34	0.01929
ENSG00000211812	TRAV26-2	−1.272	0.2803	0.7024	−1.811	0.07015
ENSG00000213003	BTF3P12	1.8782	6.5417	0.6844	2.744	0.00607
ENSG00000231150	RP1-207H1.3	−1.9134	0.1476	0.6741	−2.839	0.00453
ENSG00000236047	AC073410.1	−1.0926	0.3354	0.6397	−1.708	0.08764
ENSG00000239224	RN7SL546P	−2.1814	0.1129	0.8391	−2.6	0.00933

### Evaluate the predictive power and accuracy of the risk score in MIBC patients who received GC regimens

**Figure 4:**
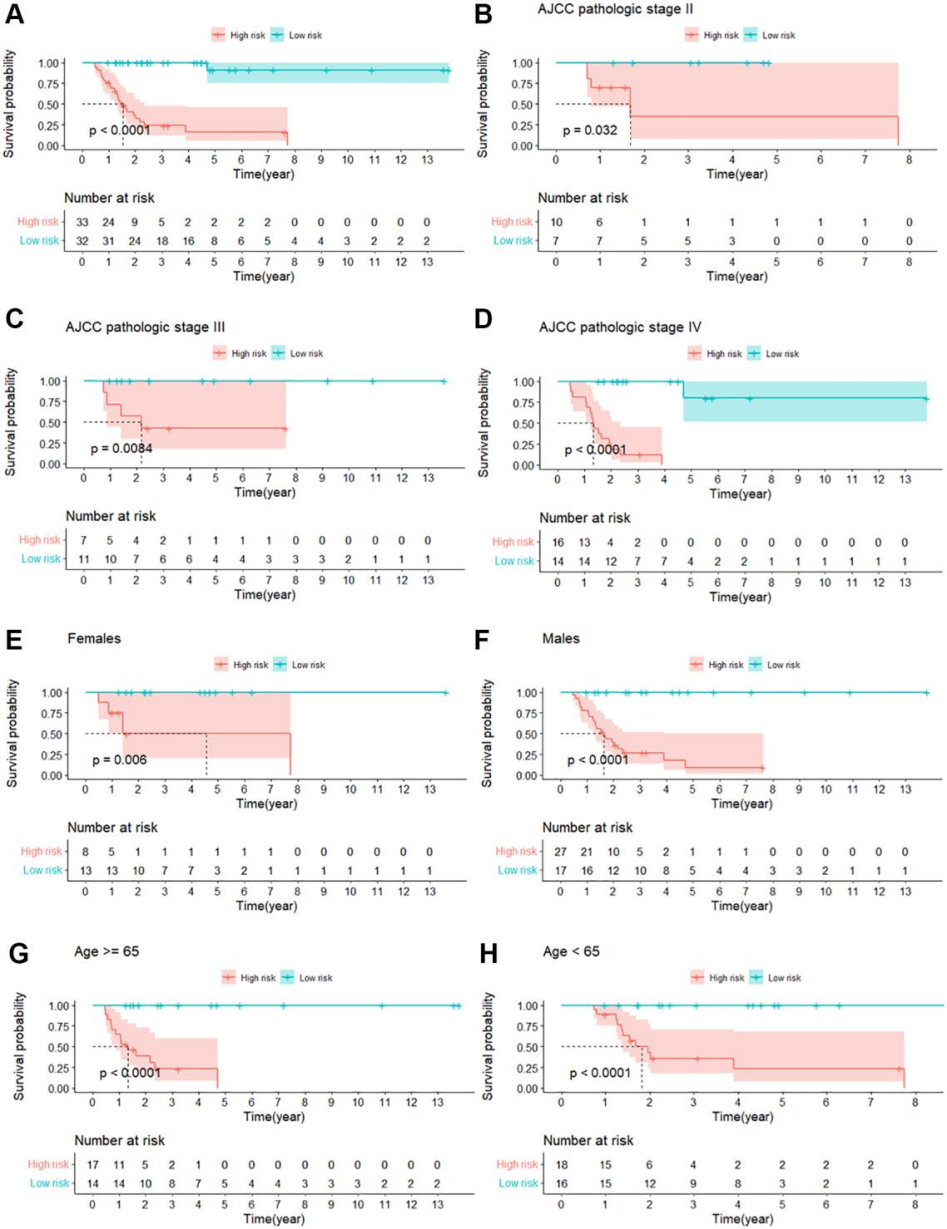
**The Kaplan–Meier survival curve between the high-risk and low-risk groups of MIBC patients who received GC regimens.** (**A**) Kaplan–Meier survival curve for the entire dataset; (**B**) Kaplan-Meier survival curve for AJCC pathology stage II; (**C**) Kaplan-Meier survival curve for AJCC pathology stage III; (**D**) Kaplan-Meier survival curve for AJCC pathology stage IV; (**E**) Kaplan-Meier survival curve for females; (**F**) Kaplan-Meier survival curve for males; (**G**) Kaplan-Meier survival curve for age ≥65; (**H**) Kaplan-Meier survival curve for age <65.

**Figure 5:**
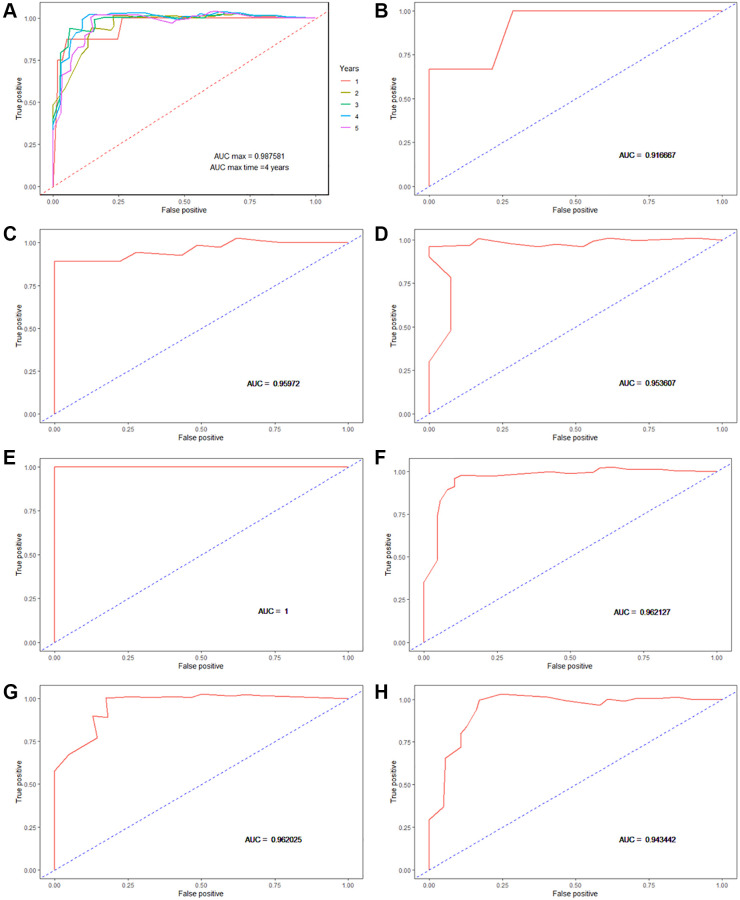
**Time-dependent receiver operating characteristic curves (ROC) and area under the curve (AUC) for OS prediction.** (**A**) Time-dependent ROC and AUC for the entire dataset; (**B**) Time-dependent ROC and AUC for AJCC pathology stage II; (**C**) Time-dependent ROC and AUC for AJCC pathology stage III; (**D**) Time-dependent ROC and AUC for AJCC pathology stage IV; (**E**) Time-dependent ROC and AUC for females; (**F**) Time-dependent ROC and AUC for males; (**G**) Time-dependent ROC and AUC for age ≥ 65; (**H**) Time-dependent ROC and AUC for age <65.

65 MIBC patients were divided into high-risk and low-risk groups by the median risk score. The Kaplan–Meier survival curve showed that patients in the high-risk group have significantly poorer OS than those in the low-risk group. (log-rank test chi-square = 38.08, *p* = 7e-10, C-index = 0.785, se = 0.032). (Figure 4A). Time-dependent ROC curves were plotted to evaluate the predictive power of the risk score in the OS prediction of 1–5 years. The time-dependent ROC curves showed that the risk score performed best in the OS prediction of 4 years (AUC = 0.987581) (Figure 5A). Further, we plotted the Kaplan–Meier survival curve in the subsets of clinical characteristics (age at diagnosis, gender, and ACJJ pathological stage) and got consistent results with the entire dataset (Figure 4B–4H, Figure 5B–5H) (Table 3). Besides, the risk score got the high C-indexes in the entire dataset and the subsets of clinical characteristics (Table 3).

**Table 3:** Kaplan-Meier survival analysis in subsets of clinical characteristics.

**Clinical characteristics**	***N***	**High-risk/Low-risk**	**Log-rank**	***P*-value**	**C-index**
Gender					
Female	21	10/11	7.56	0.006	0.861
Male	44	25/19	24.32	8e-07	0.767
Age at diagnosis (years)					
≥65	31	17/14	19.25	1e-05	0.8
<65	34	16/18	18.85	1e-05	0.811
AJCC pathological stage					
Stage II	17	10/7	4.62	0.03	0.76
Stage III	18	7/11	6.95	0.008	0.839
Stage IV	30	18/12	25.94	4e-07	0.805

### The relationship between the risk score and the clinical characteristics

**Figure 6:**
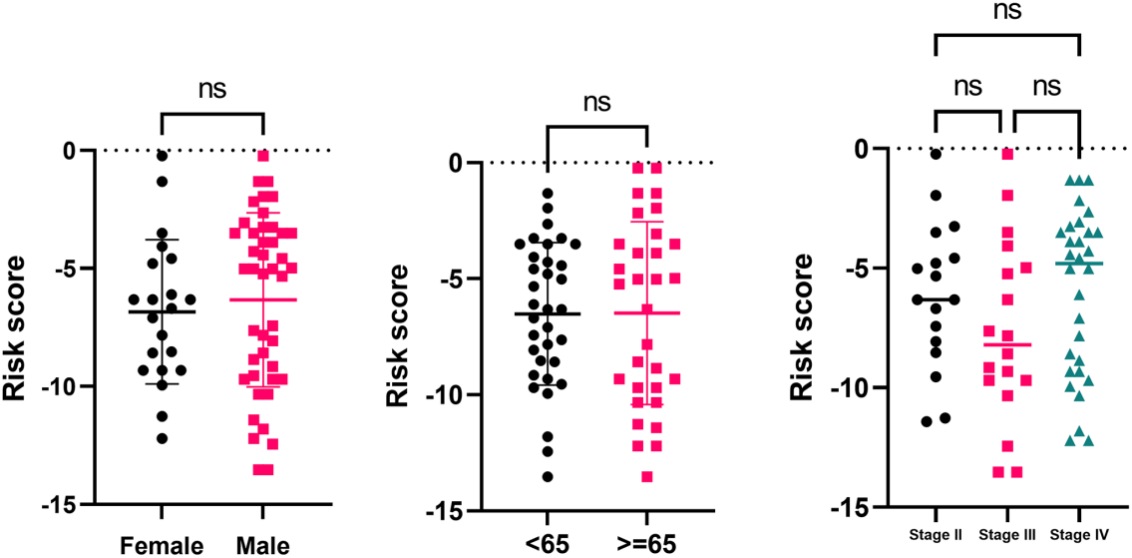
Risk score comparison in subsets of the clinical characteristics (gender, age at diagnosis, and AJCC pathological stage).

**Figure 7:**
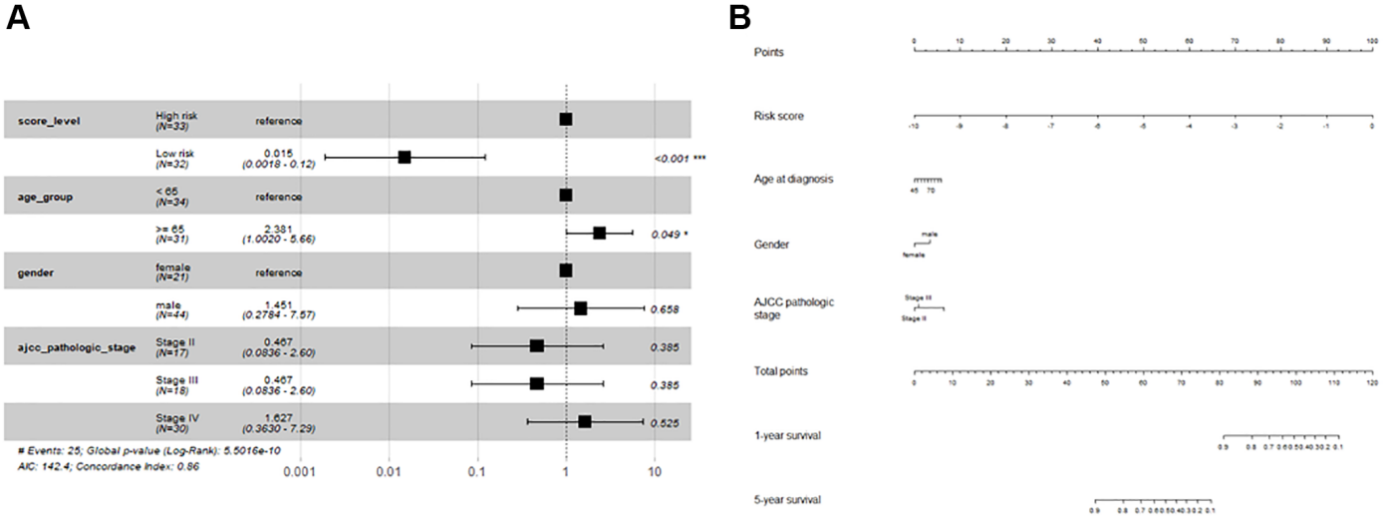
**Forest plot and nomogram for multivariate Cox regression analysis.** (**A**) Forest plot; (**B**) Nomogram.

The relationship between the risk score and the clinical characteristics (gender, age at diagnosis, and AJCC pathological stage) was investigated. The risk score was independent of gender, age at diagnosis, and AJCC pathological stage (Table 4) (Figure 6). A multivariate cox analysis and a corresponding nomogram based on risk score, gender, age at diagnosis, and AJCC pathological stage showed that risk score was an essential indicator for OS prediction, while gender, age at diagnosis, and AJCC pathological stage were not significantly associated with OS (Figure 7A, 7B).

**Table 4:** Risk score comparison in subsets of the clinical characteristics.

**Clinical characteristics**	***N***	**Mean ± sd**	**t/F**	***P*-value**
Gender				
Female	21	−5.26 ± 2.44	−1.11	0.271
Male	44	−4.51 ± 2.59		
Age at diagnosis (years)				
<65	34	−4.76 ± 2.36	−0.04	0.967
≥65	31	−4.74 ± 2.78		
AJCC pathological stage				
Stage II	17	−4.28 ± 2.41	1.33	0.272
Stage III	18	−5.56 ± 2.84		
Stage IV	30	−4.53 ± 2.41		

### Analysis of DEGs between high-risk and low-risk groups in MIBC patients who received GC regimens

**Figure 8:**
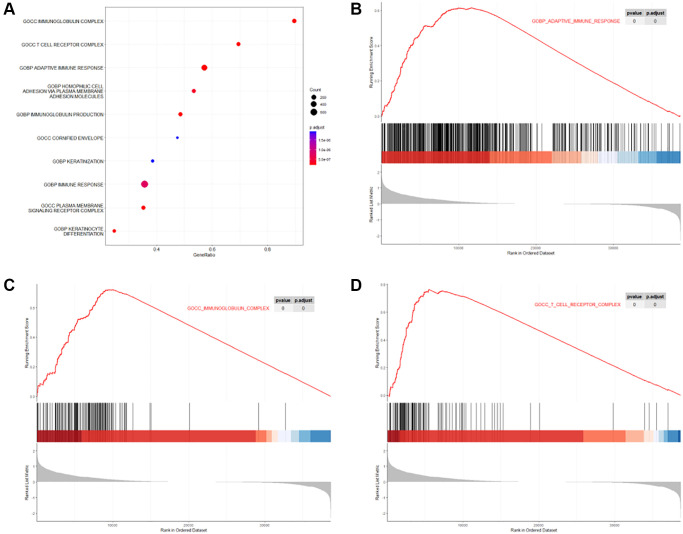
**GSEA of DEGs between high-risk and low-risk levels in MIBC patients who received GC regimens.** (**A**) Top 10 GSEA terms of the DEGs; (**B**) GSEA plot of the term “GOBP_ADAPTIVE_IMMUNE_RESPONSE”; (**C**) GSEA plot of the term “GOCC_IMMUNOGLOBULIN_COMPLEX”; (**D**) GSEA plot of term “GOCC_T_CELL_RECEPTOR_COMPLEX”.

1849 DEGs were recognized by comparing gene expression between high-risk and low-risk groups in MIBC patients who received GC regimens, among which 1463 DEGs were up-regulated, and 386 DEGs were down-regulated. These 1849 DEGs were then submitted to GSEA, and the result showed the DEGs were primarily enriched in titles related to immune processes, such as “GOBP_ADAPTIVE_IMMUNE_RESPONSE”, “GOCC_IMMUNOGLOBULIN_COMPLEX”, “GOCC_T_CELL_RECEPTOR_COMPLEX” (Figure 8).

## DISCUSSION

MIBC patients usually have poor prognoses. In clinical practice, chemotherapy in conjunction with RC is the classical treatment to improve the OS. Cisplatin-based neoadjuvant chemotherapy (NAC), for its low toxicity and high OS, is recommended as the standard MIBC chemotherapy regimen by the American Urological Association [4] and the European Association [13]; however, some topics are still worth discussing. First, in actual practice, around 19% of all patients undergo NAC before RC [20], while the remaining 81% do not for various reasons. As a result, clinicians adopt adjuvant chemotherapy (AC) after RC as an alternative. Wosnitzer MS et al. [21], Matsubara N et al. [22], and Bae WK et al. [23] all concluded no statistically significant difference between NAC and AC, while Bene G Del et al. [24] found that NAC was superior to AC in terms of disease-free survival (DFS). According to Berg et al., the survival effect of AC was only seen in individuals with pure urothelial carcinoma [25]. However, all these researches are retrospective. Macleod LC et al. [26] found significant treatment selection bias in RC timing, limiting the capacity to distinguish between NAC and AC efficacy using observational data. Patients with a higher propensity to receive NAC were expected to have a longer survival time. NAC was associated with a modest but significant survival benefit in healthier, younger patients once selection bias was considered. These findings are still expected to be validated by a prospective randomized experiment. Second, NAC/AC is not eligible for all MIBC patients. In a retrospective analysis of MIBC patients who received either NAC with RC or RC alone, Bhindi Bet et al. [27] discovered that patients who received NAC had inferior disease control and survival when their malignancy remained after chemotherapy. Third, although GC and MVAC regimens are the most used chemotherapy regimens in NAC/AC, some clinicians prefer the GC regimens to the MVAC regimens for their low toxicity and ease of administration, so how identifying individuals who would benefit from the GC regimens is a problem. In summary, it is vital to assess MIBC patients’ state to determine if they would benefit from GC regimens chemotherapy. However, relevant research was rare.

In recent years, high-throughput sequencing (HTS) technologies have generated massive amounts of gene expression data about MIBC. Previous studies have investigated the potential predictive value of the genes associated with MIBC in terms of progression and prognosis [28–34], but none has looked into the potential predictive value of the genes related to MIBC patients who received GC regimens in terms of progression and prognosis.

In this study, we developed a risk score to assess MIBC patients and to offer chemotherapy advice. The risk score was calculated by the formula: risk score = (1.3056 × ENSG00000033100) + (−1.2720 × ENSG00000211812) + (1.8782 × ENSG00000213003) + (−1.9134 × ENSG00000231150) + (−1.0926 × ENSG00000236047) + (−2.1814 × ENSG00000239224). After dividing the MIBC patients into high-risk and low-risk groups by the median risk score, we found high-risk patients had a poorer OS than low-risk patients in the entire dataset and subsets of clinical characteristics (age at diagnosis, gender, and ACJJ pathological stage). Time-dependent ROC and C-index further evaluated the risk score. The results showed that the risk score could predict the survival outcomes of MIBC patients who received GC regimens accurately and steadily in the entire dataset and subsets of clinical characteristics (age at diagnosis, gender, and ACJJ pathological stage). By analyzing the relationship between the risk score and the clinical characteristics, we found that the risk score is independent of the general clinical characteristics and a good indicator of chemotherapy management. Our risk score is useful for assessing MIBC patients’ state when clinicians advise chemotherapy for GC regimens.

In this study, we recognized 1854 survival-associated genes. We found these survival-associated genes were more enriched in terms about immunology, such as “positive regulation of natural killer cell mediated immunity” and “positive regulation of cell killing” in the GO enrichment analysis, and pathway terms about immunology, “Natural killer cell-mediated cytotoxicity”, in the KEGG enrichment analysis. While in the GSEA on DEGs between high-risk and low-risk levels, the 1849 DEGs were also more enriched in terms about immunology, such as “GOBP_ADAPTIVE_IMMUNE_RESPONSE”, “GOCC_IMMUNOGLOBULIN_COMPLEX”, “GOCC_T_CELL_RECEPTOR_COMPLEX”. Many studies have reported that immune-related genes play an important role in bladder cancer prognosis [31, 35–38], which is consistent with our findings. Besides, among the genes in the formula of the risk score, CHPF2, TRAV26-2, and BTF3P12 are found in the C7: immunologic signature gene sets downloaded from the MSigDB website, that implies the risk score reflects the immunological mechanism in the GC regimens chemotherapy of MIBC and its predictive ability may be due to introducing immune-related genes. As research on the molecular mechanisms of CHPF2, TRAV26-2, and BTF3P12 is rare, further studies are looking forward. On the other hand, when we performed GO enrichment analysis and KEGG enrichment analysis on the survival-associated genes of the patients in different ACJJ stages, the results differed from that of the entire dataset. Because of this study’s small sample size, we remain cautious about these results and look forward to further research.

We acknowledge some limitations of this study. First, we found that only the TCGA database offered the complete chemotherapeutic information we needed in this study, and the study dataset we used contained 65 patients. As a result, we developed the risk score on a small-size dataset, and we could not validate it against a valid external dataset. Next step, we will recruit additional patients to participate in our study to update or validate the risk score. Second, we found that the prognosis of MIBC patients who received GC regimens is associated with immune-related genes, where further biological experiments are warranted to validate their functions in bladder cancer. Third, our study is retrospective. Prospective randomized clinical trials are required.

In summary, we developed a risk score for the prognosis prediction of MIBC patients who received GC regimens based on the TCGA-BLCA gene profile data. The risk score was confirmed to be an independent prognostic indicator for MIBC patients who received GC regimens and potential immunotherapeutic targets for MIBC.

## References

[1] Bray F, Ferlay J, Soerjomataram I, Siegel RL, Torre LA, Jemal A. Global cancer statistics 2018: GLOBOCAN estimates of incidence and mortality worldwide for 36 cancers in 185 countries. CA Cancer J Clin. 2018; 68:394–424. 10.3322/caac.21492 30207593

[2] Saginala K, Barsouk A, Aluru JS, Rawla P, Padala SA, Barsouk A. Epidemiology of Bladder Cancer. Med Sci (Basel). 2020; 8:15. 10.3390/medsci8010015 32183076 PMC7151633

[3] Noone AM, Howlader N, Krapcho M, Miller D, Brest A, Yu M, Ruhl J, Tatalovich Z, Mariotto A, Lewis DR, Chen HS, Feuer EJ, Cronin KA. SEER Cancer Statistics Review, 1975-2015. Natl Cancer Inst. 2018. https://seer.cancer.gov/csr/1975_2015/.

[4] Chang SS, Bochner BH, Chou R, Dreicer R, Kamat AM, Lerner SP, Lotan Y, Meeks JJ, Michalski JM, Morgan TM, Quale DZ, Rosenberg JE, Zietman AL, Holzbeierlein JM. Treatment of Non-Metastatic Muscle-Invasive Bladder Cancer: AUA/ASCO/ASTRO/SUO Guideline. J Urol. 2017; 198:552–9. 10.1016/j.juro.2017.04.086 28456635 PMC5626446

[5] Malmström PU, Rintala E, Wahlqvist R, Hellström P, Hellsten S, Hannisdal E. Five-year followup of a prospective trial of radical cystectomy and neoadjuvant chemotherapy: Nordic Cystectomy Trial I. The Nordic Cooperative Bladder Cancer Study Group. J Urol. 1996; 155:1903–6. 8618283

[6] Tsushima T, Murakami T, Ebara S, Kusaka N, Kaku S, Miyaji Y, Yamamoto Y, Nasu Y, Kumon H, Ohmori H. Partial cystectomy in the treatment of invasive bladder cancer. Nishinihon J Urol. 1998; 60:397–401. https://okayama.pure.elsevier.com/en/publications/partial-cystectomy-in-the-treatment-of-invasive-bladder-cancer.

[7] Sherif A, Rintala E, Mestad O, Nilsson J, Holmberg L, Nilsson S, Malmström PU, and Nordic Urothelial Cancer Group. Neoadjuvant cisplatin-methotrexate chemotherapy for invasive bladder cancer--Nordic cystectomy trial 2. Scand J Urol Nephrol. 2002; 36:419–25. 10.1080/003655902762467567 12623505

[8] Griffiths G, Hall R, Sylvester R, Raghavan D, Parmar MK, and International Collaboration of Trialists, and Medical Research Council Advanced Bladder Cancer Working Party (now the National Cancer Research Institute Bladder Cancer Clinical Studies Group), and European Organisation for Research and Treatment of Cancer Genito-Urinary Tract Cancer Group, and Australian Bladder Cancer Study Group, and National Cancer Institute of Canada Clinical Trials Group, and Finnbladder, and Norwegian Bladder Cancer Study Group, and Club Urologico Espanol de Tratamiento Oncologico Group. International phase III trial assessing neoadjuvant cisplatin, methotrexate, and vinblastine chemotherapy for muscle-invasive bladder cancer: long-term results of the BA06 30894 trial. J Clin Oncol. 2011; 29:2171–7. 10.1200/JCO.2010.32.3139 21502557 PMC3107740

[9] Choueiri TK, Jacobus S, Bellmunt J, Qu A, Appleman LJ, Tretter C, Bubley GJ, Stack EC, Signoretti S, Walsh M, Steele G, Hirsch M, Sweeney CJ, et al. Neoadjuvant dose-dense methotrexate, vinblastine, doxorubicin, and cisplatin with pegfilgrastim support in muscle-invasive urothelial cancer: pathologic, radiologic, and biomarker correlates. J Clin Oncol. 2014; 32:1889–94. 10.1200/JCO.2013.52.4785 24821883 PMC7057274

[10] Plimack ER, Hoffman-Censits JH, Viterbo R, Trabulsi EJ, Ross EA, Greenberg RE, Chen DY, Lallas CD, Wong YN, Lin J, Kutikov A, Dotan E, Brennan TA, et al. Accelerated methotrexate, vinblastine, doxorubicin, and cisplatin is safe, effective, and efficient neoadjuvant treatment for muscle-invasive bladder cancer: results of a multicenter phase II study with molecular correlates of response and toxicity. J Clin Oncol. 2014; 32:1895–901. 10.1200/JCO.2013.53.2465 24821881 PMC4050203

[11] Advanced Bladder Cancer (ABC) Meta-analysis Collaboration. Adjuvant chemotherapy in invasive bladder cancer: a systematic review and meta-analysis of individual patient data Advanced Bladder Cancer (ABC) Meta-analysis Collaboration. Eur Urol. 2005; 48:189–99. 10.1016/j.eururo.2005.04.005 15939530

[12] Yin M, Joshi M, Meijer RP, Glantz M, Holder S, Harvey HA, Kaag M, Fransen van de Putte EE, Horenblas S, Drabick JJ. Neoadjuvant Chemotherapy for Muscle-Invasive Bladder Cancer: A Systematic Review and Two-Step Meta-Analysis. Oncologist. 2016; 21:708–15. 10.1634/theoncologist.2015-0440 27053504 PMC4912364

[13] Witjes JA, Bruins HM, Cathomas R, Compérat EM, Cowan NC, Gakis G, Hernández V, Linares Espinós E, Lorch A, Neuzillet Y, Rouanne M, Thalmann GN, Veskimäe E, et al. European Association of Urology Guidelines on Muscle-invasive and Metastatic Bladder Cancer: Summary of the 2020 Guidelines. Eur Urol. 2021; 79:82–104. 10.1016/j.eururo.2020.03.055 32360052

[14] Galsky MD, Pal SK, Chowdhury S, Harshman LC, Crabb SJ, Wong YN, Yu EY, Powles T, Moshier EL, Ladoire S, Hussain SA, Agarwal N, Vaishampayan UN, et al, and Retrospective International Study of Cancers of the Urothelial Tract (RISC) Investigators. Comparative effectiveness of gemcitabine plus cisplatin versus methotrexate, vinblastine, doxorubicin, plus cisplatin as neoadjuvant therapy for muscle-invasive bladder cancer. Cancer. 2015; 121:2586–93. 10.1002/cncr.29387 25872978

[15] Goel S, Sinha RJ, Bhaskar V, Aeron R, Sharma A, Singh V. Role of gemcitabine and cisplatin as neoadjuvant chemotherapy in muscle invasive bladder cancer: Experience over the last decade. Asian J Urol. 2019; 6:222–9. 10.1016/j.ajur.2018.06.006 31297313 PMC6595093

[16] Durinck S, Spellman PT, Birney E, Huber W. Mapping identifiers for the integration of genomic datasets with the R/Bioconductor package biomaRt. Nat Protoc. 2009; 4:1184–91. 10.1038/nprot.2009.97 19617889 PMC3159387

[17] Wu T, Hu E, Xu S, Chen M, Guo P, Dai Z, Feng T, Zhou L, Tang W, Zhan L, Fu X, Liu S, Bo X, Yu G. clusterProfiler 4.0: A universal enrichment tool for interpreting omics data. Innovation (Camb). 2021; 2:100141. 10.1016/j.xinn.2021.100141 34557778 PMC8454663

[18] Friedman J, Hastie T, Tibshirani R. Regularization Paths for Generalized Linear Models via Coordinate Descent. J Stat Softw. 2010; 33:1–22. 20808728 PMC2929880

[19] Chen Y, Lun AT, Smyth GK. From reads to genes to pathways: differential expression analysis of RNA-Seq experiments using Rsubread and the edgeR quasi-likelihood pipeline. F1000Res. 2016; 5:1438. 10.12688/f1000research.8987.2 27508061 PMC4934518

[20] Hanna N, Trinh QD, Seisen T, Vetterlein MW, Sammon J, Preston MA, Lipsitz SR, Bellmunt J, Menon M, Choueiri TK, Abdollah F. Effectiveness of Neoadjuvant Chemotherapy for Muscle-invasive Bladder Cancer in the Current Real World Setting in the USA. Eur Urol Oncol. 2018; 1:83–90. 10.1016/j.euo.2018.03.001 31100232

[21] Wosnitzer MS, Hruby GW, Murphy AM, Barlow LJ, Cordon-Cardo C, Mansukhani M, Petrylak DP, Benson MC, McKiernan JM. A comparison of the outcomes of neoadjuvant and adjuvant chemotherapy for clinical T2-T4aN0-N2M0 bladder cancer. Cancer. 2012; 118:358–64. 10.1002/cncr.26278 21717438

[22] Matsubara N, Mukai H, Naito Y, Nezu M, Itoh K. Comparison between neoadjuvant and adjuvant gemcitabine plus cisplatin chemotherapy for muscle-invasive bladder cancer. Asia Pac J Clin Oncol. 2013; 9:310–7. 10.1111/ajco.12017 23127231 PMC3933765

[23] Bae WK, Lee HJ, Park SH, Kim JH, Kim HJ, Maeng CH, Park I, Sohn BS, Kim JA, Lee KH, Lim DH, Chang H, Kim SM, et al. Comparative effectiveness of palliative chemotherapy versus neoadjuvant chemotherapy followed by radical cystectomy versus cystectomy followed by adjuvant chemotherapy versus cystectomy for regional node-positive bladder cancer: A retrospective analysis: KCSG GU 17-03. Cancer Med. 2019; 8:5431–7. 10.1002/cam4.2446 31353788 PMC6745843

[24] Del Bene G, Calabrò F, Giannarelli D, Plimack ER, Harshman LC, Yu EY, Crabb SJ, Pal SK, Alva AS, Powles T, De Giorgi U, Agarwal N, Bamias A, et al. Neoadjuvant vs. Adjuvant Chemotherapy in Muscle Invasive Bladder Cancer (MIBC): Analysis From the RISC Database. Front Oncol. 2018; 8:463. 10.3389/fonc.2018.00463 30510914 PMC6252384

[25] Berg S, D'Andrea D, Vetterlein MW, Cole AP, Fletcher SA, Krimphove MJ, Marchese M, Lipsitz SR, Sonpavde G, Noldus J, Shariat SF, Kibel AS, Trinh QD, Mossanen M. Impact of adjuvant chemotherapy in patients with adverse features and variant histology at radical cystectomy for muscle-invasive carcinoma of the bladder: Does histologic subtype matter? Cancer. 2019; 125:1449–58. 10.1002/cncr.31952 30620387

[26] Macleod LC, Fam MM, Yabes JG, Hale NE, Turner RM 2nd, Lopa SH, Gingrich JR, Borza T, Skolarus TA, Davies BJ, Jacobs BL. Comparison of Neoadjuvant and Adjuvant Chemotherapy in Muscle-invasive Bladder Cancer. Clin Genitourin Cancer. 2020; 18:201–9.e2. 10.1016/j.clgc.2019.12.011 31917172

[27] Bimal B, Igor F, Ross M, Robert FT, Prabin T, John CC, Brian AC, Lance CP, Jeffrey K, Robert HT, Matthew KT, Stephen AB. Survival for patients with residual tumor at radical cystectomy following chemotherapy: A matched analysis to cystectomy-only patients. J Clin Oncol. 2017; 35:355. 10.1200/JCO.2017.35.6_SUPPL.355

[28] Xu Y, Wu G, Li J, Li J, Ruan N, Ma L, Han X, Wei Y, Li L, Zhang H, Chen Y, Xia Q. Screening and Identification of Key Biomarkers for Bladder Cancer: A Study Based on TCGA and GEO Data. Biomed Res Int. 2020; 2020:8283401. 10.1155/2020/8283401 32047816 PMC7003274

[29] Wang Y, Chen L, Ju L, Qian K, Liu X, Wang X, Xiao Y. Novel Biomarkers Associated With Progression and Prognosis of Bladder Cancer Identified by Co-expression Analysis. Front Oncol. 2019; 9:1030. 10.3389/fonc.2019.01030 31681575 PMC6799077

[30] Luo Y, Zeng G, Wu S. Identification of Microenvironment-Related Prognostic Genes in Bladder Cancer Based on Gene Expression Profile. Front Genet. 2019; 10:1187. 10.3389/fgene.2019.01187 31824575 PMC6883806

[31] Zhu J, Wang H, Ma T, He Y, Shen M, Song W, Wang JJ, Shi JP, Wu MY, Liu C, Wang WJ, Huang YQ. Identification of immune-related genes as prognostic factors in bladder cancer. Sci Rep. 2020; 10:19695. 10.1038/s41598-020-76688-w 33184436 PMC7661532

[32] Zhu R, Yang X, Guo W, Xu XJ, Zhu L. An eight-mRNA signature predicts the prognosis of patients with bladder urothelial carcinoma. PeerJ. 2019; 7:e7836. 10.7717/peerj.7836 31660264 PMC6814068

[33] Cao R, Yuan L, Ma B, Wang G, Qiu W, Tian Y. An EMT-related gene signature for the prognosis of human bladder cancer. J Cell Mol Med. 2020; 24:605–17. 10.1111/jcmm.14767 31657881 PMC6933372

[34] Chu J, Li N, Li F. A risk score staging system based on the expression of seven genes predicts the outcome of bladder cancer. Oncol Lett. 2018; 16:2091–6. 10.3892/ol.2018.8904 30008905 PMC6036497

[35] Chen X, Jin Y, Gong L, He D, Cheng Y, Xiao M, Zhu Y, Wang Z, Cao K. Bioinformatics Analysis Finds Immune Gene Markers Related to the Prognosis of Bladder Cancer. Front Genet. 2020; 11:607. 10.3389/fgene.2020.00607 32655621 PMC7324668

[36] Cao J, Yang X, Li J, Wu H, Li P, Yao Z, Dong Z, Tian J. Screening and Identifying Immune-Related Cells and Genes in the Tumor Microenvironment of Bladder Urothelial Carcinoma: Based on TCGA Database and Bioinformatics. Front Oncol. 2020; 9:1533. 10.3389/fonc.2019.01533 32010623 PMC6974676

[37] Jin K, Qiu S, Jin D, Zhou X, Zheng X, Li J, Liao X, Yang L, Wei Q. Development of prognostic signature based on immune-related genes in muscle-invasive bladder cancer: bioinformatics analysis of TCGA database. Aging (Albany NY). 2021; 13:1859–71. 10.18632/aging.103787 33465047 PMC7880322

[38] Wu X, Lv D, Cai C, Zhao Z, Wang M, Chen W, Liu Y. A TP53-Associated Immune Prognostic Signature for the Prediction of Overall Survival and Therapeutic Responses in Muscle-Invasive Bladder Cancer. Front Immunol. 2020; 11:590618. 10.3389/fimmu.2020.590618 33391264 PMC7774015

